# Clinical Course of Dual-Chamber Implantable Cardioverter-Defibrillator Recipients followed by Cardiac Remote Monitoring: Insights from the LION Registry

**DOI:** 10.1155/2018/3120480

**Published:** 2018-11-04

**Authors:** Joerg O. Schwab, Herbert Nägele, Hanno Oswald, Gunnar Klein, Oliver Gunkel, Andreas Lang, Wolfgang R. Bauer, Paul Korb, Tino Hauser

**Affiliations:** ^1^Department of Therapeutic and Interventional Cardiology, Beta Clinic, The International Hospital, Bonn, Germany; ^2^Hospital Reinbek St. Adolf Stift, Reinbek, Germany; ^3^Hannover Medical School, Hannover, Germany; ^4^Hospital Frankfurt/Oder, Frankfurt/Oder, Germany; ^5^Joint Practice of Internistic and Cardiology, Erfurt, Germany; ^6^University Hospital Würzburg, Würzburg, Germany; ^7^Biotronik, Berlin, Germany

## Abstract

Patients receiving dual-chamber implantable cardioverter-defibrillator (DR-ICD) therapy are at risk of developing atrial arrhythmia because of the increased rate of ventricular pacing and the progression of heart failure. Remote monitoring (RM) may identify the patients at highest risk of adverse events such as atrial arrhythmias. A total of 283 patients with 91,632 remote transmissions during a 15-month follow-up (FU) period enrolled in the LION registry were analysed. The parameters retrieved included the pacing mode, lower rate limit, percentage of atrial (%AP) and ventricular pacing (%VP), and percentage of atrial arrhythmia burden (%AB). In 92.7% of patients, the devices were initially programmed in DDD(R) or DDI(R), with changes of the pacing mode in 19.3% only. The lower rate limit remained stable in 80.4% of patients. At the first transmission, 8.7% of patients suffered from RM-detected atrial arrhythmia, which reached 36% during FU. The %AP was not associated with increased AB (*p* = 0.67), but the %VP was different in patients developing RM-detected atrial arrhythmia (26.9% vs. 13.7%,* p* < 0.00001). The %VP increased in 105 patients (significance level of *α* = 0.05), and 11 patients crossed the border of 50% VP. The LION substudy supports the concept of using RM in a real-world DR-ICD population. Remote monitoring of DR-ICDs allows for the quantification of the course of the pacing parameters and AB. Based on these observations, device parameters can be adjusted and optimized.

## 1. Introduction

Implantable cardioverter-defibrillator (ICD) therapy has become the standard of care for the primary and secondary prevention of sudden cardiac death [1, 2]. In patients with heart failure, ICD or cardiac resynchronization therapy (CRT) has proven to be beneficial by significantly reducing mortality and rehospitalization for heart failure and other major cardiovascular events [3, 4].

In patients with dual chamber devices, ventricular dyssynchronization often occurs as a result of chronic right ventricular pacing, and it may promote the progression of heart failure with subsequent rehospitalization, atrial fibrillation (AF), and mortality [5–8]. Atrial fibrillation reflects a major safety concern with ICD implantation as it is associated with an increased risk of stroke and mortality [9, 10]. Limited data are available about pacemaker-related indications for dual-chamber ICDs as well as the underlying pacing requirements at the time of dual-chamber ICD implantation and during follow-up under real world conditions [11, 12].

As the incidence of electrophysiologic disturbances increases with the aging of the population, remote monitoring has become an attractive alternative to in-clinic follow-up visits, which are one of the most frequent and expensive activities performed in electrophysiology services [13]. Based on the latest iteration of the European Society of Cardiology guidelines on cardiac pacing and resynchronization, remote monitoring is a class IIa recommendation for patients with implanted devices [14]. Moreover, an HRS Expert Consensus Statement recommends that remote monitoring should be offered to all patients with cardiovascular implantable electronic devices (class I recommendation, level of evidence A) [15]. This approach allows the rapid detection of arrhythmic events, the optimisation of medical treatment, and device programming, while necessitating low healthcare resource consumption [16–19]. In ICD and CRT-D patients with heart failure, the IN-TIME trial revealed that those who were followed by implant-based telemonitoring with daily transmission demonstrated a reduced risk of worsening heart failure and, moreover, all-cause mortality [20].

In the present study, we have investigated the clinical course of dual-chamber ICD recipients in a subset of patients enrolled in the LION trial with activated automatic remote monitoring. The specific objectives were to evaluate the atrial and ventricular pacing, the changes in pacemaker settings, and the association between atrial and ventricular pacing and the development of atrial arrhythmias. These parameters were retrieved from the remote monitoring data set of the LION trial which provided much more data points, e.g., regarding atrial and ventricular pacing compared to conventional device interrogations during on-site follow-ups.

## 2. Methods

### 2.1. Study Design and Patient Selection

The LION registry was a prospective, nonrandomised, multicentre trial which enrolled 1533 patients with an indication for an ICD or a CRT defibrillator according to current guidelines [1, 14]. The aim of the study was to assess the percentage of correct classification of IEGM Online HD in a real-world setting. The study design, methods, and primary results have been published recently [21]. Briefly, 1530 patients with implanted devices either received a single-chamber ICD (n = 717), a dual-chamber ICD (n = 361) or a CRT defibrillator (n = 452) from the Lumax® family (Biotronik SE & Co. KG, Berlin, Germany) equipped with wireless Home Monitoring (HM; Biotronik SE & Co. KG, Berlin, Germany) capability. The results demonstrated that remote IEGM analysis was accurate for the classification of arrhythmic episodes. Of the 361 patients with a dual-chamber ICD, 283 were available for the subgroup analysis because they had an activated HM which provided time-triggered transmissions. Therefore, a total of over 90,000 remote transmissions were retrieved.

The study was conducted in compliance with the Good Clinical Practice guidelines and the Declaration of Helsinki. The protocol was reviewed and approved by appropriate national and local ethics committees, and all patients provided written informed consent. The use of anonymised HM data for scientific purposes beyond the original study objective is covered by the patient's informed consent and by German data protection law. All authors have read and agreed to the manuscript as written.

### 2.2. Remote Monitoring

The HM system transmits automatic daily data, such as the number of arrhythmic episodes, arrhythmia diagnostic, therapies, rhythm information, and technical parameters, via wireless telemetry to the CardioMessenger II transmitter device (Biotronik SE & Co. KG, Berlin, Germany). Data transmission is initiated once daily in a time-controlled fashion as well as upon the detection of relevant arrhythmic or technical events, and the transmitter forwards the data via a Global System for Mobile Communication to Biotronik's HM Service Centre (Berlin, Germany). The data is then decoded, stored, and placed on a password-protected internet platform which allows the patient's physician access. The HM system also sends alerts to the patient's physician via e-mail or cell phone for all prespecified events.

### 2.3. Device Programming and Study Protocol

The device programming was left to the discretion of the treating physician. The following parameters were analysed at the patient level (expressed as monthly means): the percentage of atrial pacing (% AP) defined as the number of paced atrial beats divided by the total number of atrial beats over a 24-hour period, the percentage of right ventricular pacing (% VP) defined as the number of paced ventricular beats divided by the total number of ventricular beats over a 24-hour period, the pacing mode, the lower stimulation rate, and the atrial arrhythmia burden (AB). AB was calculated as the percentage of time during a 24-hour period during which an atrial arrhythmia was detected and was defined as an atrial rhythm above the detection limit (default of 200 beats per minute).

### 2.4. Follow-Up and Data Management

Patient management was performed according to the guidelines at that time and as reported in the main paper [21]. Demographic and baseline data were collected on case report forms. The HM data for the dual-chamber ICD analysis were obtained from the HM Service Centre.

### 2.5. Statistical Analyses

Results were expressed as absolute values, percentages, means ± standard deviation, and medians with interquartile range. For AP, VP, and AB, monthly means were calculated of the daily percentage values that were reported by the HM transmissions. The resulting monthly mean % AP, mean % VP, and mean % AB were tested for a significant difference between month 1 and month 15 by a 2-sided Wilcoxon signed-rank test at a significance level of alpha = 0.05. VP was in addition tested for an intraindividual increase between the first 45 HM transmissions and the last 45 HM transmissions of each subject by two 1-sided Wilcoxon signed-rank tests at a combined significance level of 0.05 (0.025 for each 1-sided test). For AB, subgroups were established as “AB = 0”: no AB on any day during the observational period vs. “AB > 0”: AB > 0 on at least one day during the observational period. These subgroups were tested for differences in baseline parameters by t-tests and Fisher's exact tests and for an association with mean % AP and mean % VP over the entire follow-up period by a 2-sided Wilcoxon rank-sum test. All tests were evaluated at alpha = 0.05.

No adjustments for multiple testing were performed due to the exploratory nature of this investigation. No prospective sample size calculation for these analyses and no retrospective analyses of power were performed. All analyses were performed using SAS Version (9.3) (SAS Institute, Inc.).

## 3. Results

The LION registry enrolled 361 patients with a dual-chamber ICD (Lumax DR-T 300, 340, 500 and 540 models, Biotronik SE & Co. KG (Berlin, Germany)). Of those, 283 patients with 91,632 time-triggered HM transmissions met the predefined requirements. Baseline characteristics of the analysed patients are displayed in Table 1. The median HM observation period lasted 437 days (interquartile range: 390 – 445). Overall, the mean age of the patients was 65.7 ± 10.9 years, 83.4% were male, and 77.0% had no prior history of AF (based on n = 282). The mean ejection fraction was 35.4 ± 13.1% (based on n = 236).

### 3.1. Pacemaker Programming

For both the programming of the pacing mode and the lower rate limit analyses, data from 276 patients with a minimum of 45 HM transmissions were analysed. Of those, 256 patients (92.7%) had their ICD initially programmed with DDD(R) (n = 223) or DDI(R) mode (n = 33), and 80.7% (n = 223) had no change in the programmed pacing mode during follow-up. The initially programmed mean of the lower rate limit was 56 ± 13 beats per minute, and it remained stable in 80.4% of patients during follow-up.

### 3.2. Atrial Pacing during Follow-Up

**Figure 1:**
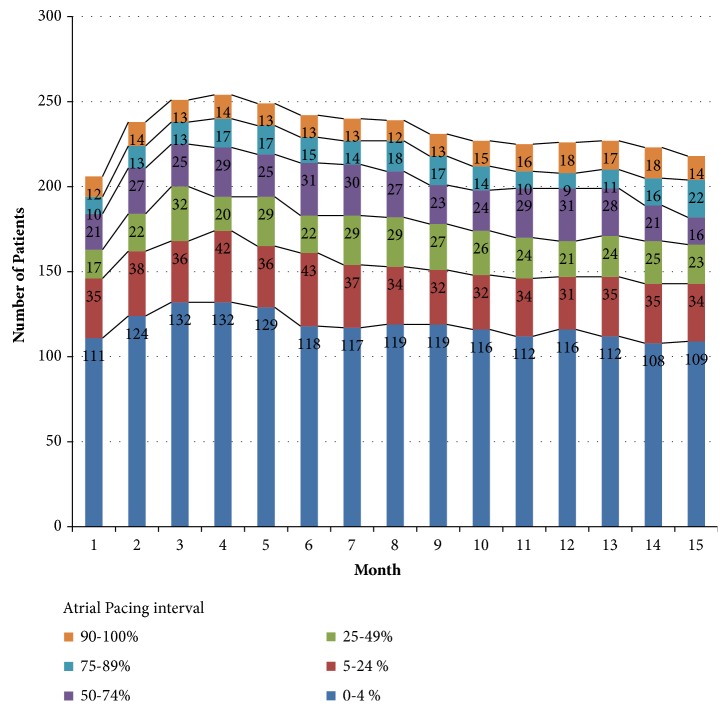
Monthly prevalence of the mean percentage of atrial pacing during follow-up.

The monthly prevalence of the mean % AP during follow-up is presented in Figure 1. More than 48% of patients had a monthly mean % AP between 0% and 4%, and less than 26% of patients had a high % AP (≥ 50 %). There was a trend of increased monthly mean % AP during the course of follow-up (from 21.5% to 25.7%), but there was no statistical difference between the month 1 (21.5% ± 31.3%) and month 15 (25.7% ± 33.8%) time points (*p* = 0.176).

### 3.3. Ventricular Pacing during Follow-Up

**Figure 2:**
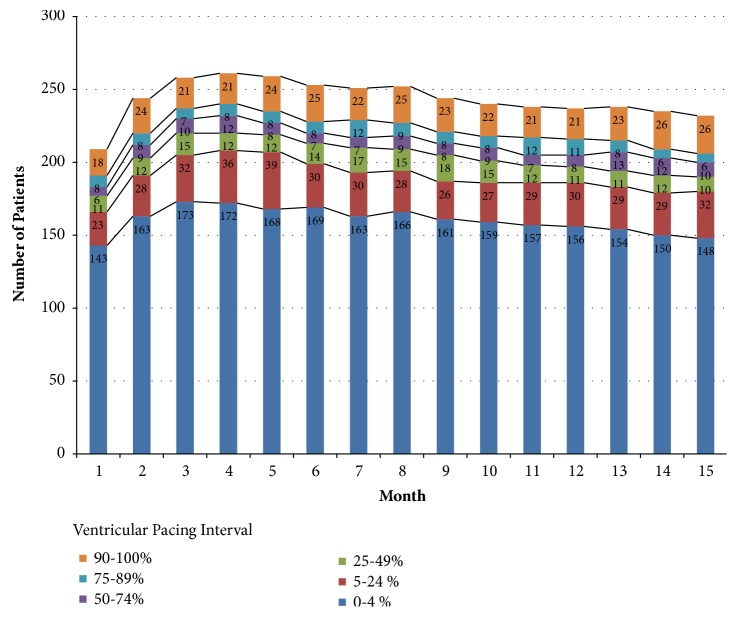
Monthly prevalence of the mean percentage of ventricular pacing during follow-up.

**Figure 3:**
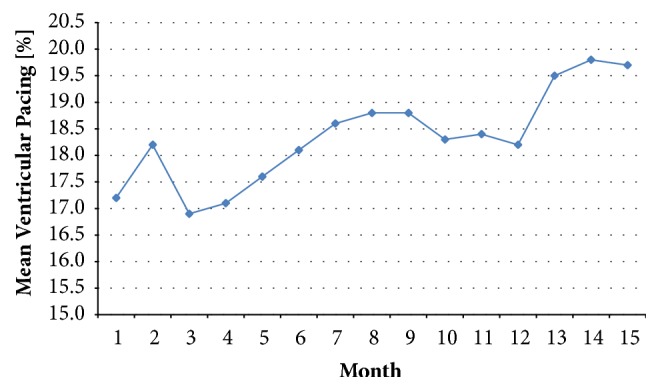
Mean percentage of ventricular pacing during follow-up.

The monthly prevalence of the mean % VP during follow-up is presented in Figure 2. More than 63% of patients had a monthly mean % VP between 0% and 4%. The monthly prevalence of % VP ≥ 50% during follow-up was between 14.7% and 18.8%. There was a trend of increasing mean % VP during the course of follow-up (Figure 3), but there was no statistical difference between the month 1 (17.2% ± 31.8%) and month 15 (19.7% ± 33.8%) time points (*p* = 0.068). The intraindividual comparison of the % VP for the first 45 HM transmissions versus the last 45 HM transmissions for 275 analysable patients revealed that 38.2% showed a significant increase in % VP (significance level of alpha = 0.05) while 24.4% had a significant decrease (significance level of alpha = 0.05). Of the 105 patients with a significant % VP increase, 10.5% crossed the border of 50% VP.

### 3.4. Atrial Arrhythmia Burden during Follow-Up

For the entire patient population, there was a trend towards increasing mean AB during the course of follow-up (from 4.4% ± 18.7% to 8.4% ± 26.9%), but there was no statistical difference between the month 1 and month 15. A total of 275 patients with a minimum of 45 HM transmissions and information about AB were divided into subgroups with no AB on any day (AB = 0) or AB > 0 on at least one day. A total of 99 patients (36%) had an AB > 0 on at least one day during the observational period.

The comparison of baseline patient parameters of these two subgroups is shown in Table 2. Subjects with AB during follo-up differed from subjects without AB during follow-up by the following characteristics (*p* < 0.05): they were more frequently in NYHA functional class ≥ III (AB = 0: 15% vs. AB > 0: 31%,* p* = 0.003), had a lower mean LVEF (AB = 0: 36.6% ± 13.6 % vs. AB > 0: 33.1% ± 12.2 %,* p* = 0.047), and had more frequently a history of atrial fibrillation (AB = 0: 13% vs. AB > 0: 40%,* p* < 0.001). Furthermore, subjects with AB during follow-up were on average 2 years older, but this trend was not significant (AB = 0: 65.1 ± 10.8 years vs. AB > 0: 67.5 ± 10.1 years;* p* = 0.071). There was no substantial difference between subjects with AB and subjects without AB during follow-up regarding gender, body mass index, sinus bradycardia, cardiomyopathy, secondary prevention indication, noncardiac medical history, and medication.

Of the 275 patients with available data about AB, there were 274 patients with additional historical information of AF occurrence. Of the 211 patients with no documented history of AF and AB data, 59 patients without a history of AF (28.0%) developed* de novo* high rate atrial tachyarrhythmias during follow-up. For 63.5% of patients with a history of AF, an AB > 0 was detected during the observation period. In contrast, no pre-existing AF history predicted the absence of device-based AF in 72.0% during the 15 months follow-up.  Table 3 summarizes the distribution of patients with available information about AB and history of AF.

### 3.5. Association between Atrial Arrhythmia Burden, Percentage of Atrial Pacing, and Percentage of Ventricular Pacing

**Figure 4:**
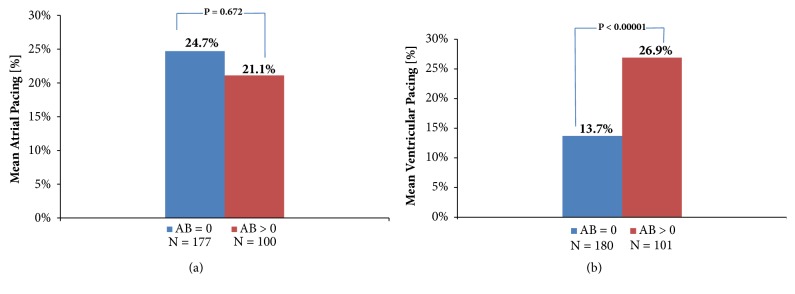
Association between the atrial arrhythmia burden, the percentage of atrial pacing, and the percentage of ventricular pacing. The comparisons are based on the mean %AP and %VP values over the entire follow-up period (the mean values are based on all available remote data transmissions of all patients in the respective AB group). Group AB = 0 includes patients who never had a transmitted AB value > 0 over the follow-up period. Group AB > 0 includes patients who had a least one AB value > 0 over the follow-up period.

In patients with an AB = 0 or AB > 0 on at least one day during follow-up, the mean % AP was not associated with an increased AB (*p* = 0.672). In contrast, the mean % VP was significantly different in patients with device-detected atrial arrhythmias (26.9% vs 13.7% those without,* p* < 0.00001) (Figure 4).

## 4. Discussion

In this analysis of over 90,000 remote HM transmissions from a large dual-chamber ICD patient registry, we found that ventricular, but not atrial pacing, was correlated with a higher incidence of device-detected atrial arrhythmias. It has been shown that right ventricular pacing increases ventricular dyssynchronization in ICD patients with existing ventricular dysfunction [5]. A correlation between the amount of right ventricular pacing and the incidence of atrial arrhythmias was found in pacemaker patients [22] which is in line with our finding where VP was correlated with the AB, with patients with AB > 0 having a higher mean % VP.

The initially programmed pacing mode and the lower stimulation rate were not modified in the majority of patients. We also found that the occurrence of device-detected high rate atrial arrhythmia episodes was predicted by a history of AF. On the other hand, no pre-existing AF history predicted the absence of AF during the course of ICD therapy. This fact might help in terms of determining possible anticoagulation therapy for device based AF. In addition, a relevant number of patients (28.0%) developed device-based atrial arrhythmias without having a history of AF. A trend for increasing mean % VP and mean % AP was also observed during follow-up, and 38% of patients had a significant % VP increase with 10.5 % crossing the border of 50 % VP.

The benefit of remote monitoring has been clearly demonstrated in numerous clinical trials [15–20]. The main results of the LION study showed the good accuracy of Home Monitoring transmitted IEGM Online for the classification of rhythm disorders as well as the clinical relevance of the remote detection of arrhythmias or sensing failures [21]. Our results extend these findings with respect to the pacing characteristics and atrial burden of a real-world dual-chamber ICD patient population.

High VP retrieved from HM messages enables rapid medical response. Because ventricular dyssynchronization often occurs as a result of chronic right ventricular pacing and may promote the progression of heart failure and the occurrence of AF and death, especially in patients presenting with a low left ventricular ejection fraction such as seen in our patient population [5, 7, 8], remote monitoring could provide early and automatic information about these dynamic changes in patients with a dual-chamber ICD. These clinically relevant events enable prompt medical responses such as optimizing device programming or to upgrade to a CRT system. Unnecessary right ventricular pacing should be minimized by using specific algorithms or programming of longer atrioventricular delays [23]. The current ESC guidelines recommend an upgrade to a CRT system (class IB) for patients with heart failure with a high percentage of ventricular pacing and additional risk factors such as LVEF < 35% [14]. The ACC/AHA/HRS guidelines recommend CRT (class IIa) for patients who have LVEF less than or equal to 35% and are undergoing new or replacement device placement with anticipated requirement for significant (> 40%) ventricular pacing [2]. Also, the continuous remote monitoring of dual-chamber ICD patients could help to identify those who would benefit more from a CRT upgrade. Indeed, we have found a relatively high percentage (36%) of dual-chamber ICD patients with device-detected atrial arrhythmia episodes during the 15-month follow-up period. Moreover, we were also able to detect that about one out of four patients without a prior history of AF developed device-detected AF following ICD implantation.

### 4.1. Study Limitations

There are several limitations to our study. First, it was a retrospective analysis of all patients implanted with a dual-chamber ICD and active remote monitoring and, as such, limits the possibility of direct comparison with other type of ICD. Second, data on the clinical pacing indications for the implantation of dual-chamber ICD were not consistently collected. Nevertheless, we believe our results are clinically relevant and should be taken into account when considering pacing therapy. Finally, the small sample size and the relatively short follow-up period may not fully confirm the effectiveness of remote monitoring in real-world ICD patients.

## 5. Conclusion

In conclusion, the LION substudy supports the concept of using remote monitoring in a real-world dual-chamber ICD population. Our results demonstrate the feasibility and usefulness of remote monitoring for the reprogramming of the pacing settings to reduce right VP and AB and provide an additional and important element for the optimisation of heart failure therapy in patients with ICD.

## Figures and Tables

**Table 1:** Baseline patient characteristics (N = 283).

Patient characteristics	N (%), mean ± SD
Male gender	236 (83)

Age (years)	65,7 ± 10,9

Body Mass Index^a^	27,6 ± 4,9

NYHA functional class	
I	28 (10)
II	106 (37)
III	56 (20)
IV	2 (1)
No heart failure	17 (6)
Missing	74 (26)

LVEF (%)^b^	35,4 ± 13,1

Secondary prevention indication	147 (52)

History of atrial fibrillation^c^	65 (23)

Ischemic^c^	186 (66)

Diabetes mellitus^c^	72 (25)

Renal insufficiency^c^	74 (26)

Sinus bradycardia^c^	44 (16)

Cardiomyopathy^d^	157 (56)

Medication^c^	

ACE inhibitor / angiotensin antagonist	234 (83)

Amiodarone	65 (23)

Beta-blocker	246 (87)

Ca-antagonist	29 (10)

Spironolactone	96 (34)

Other diuretic	188 (66)

*Abbreviations.* ACE: angiotensin-converting enzyme; NYHA: New York Heart Association; SD: standard deviation; LVEF: left ventricular ejection fraction.

^a^ 276 patients with reported data.

^b^ 236 patients with reported data.

^c^ 282 patients with reported data.

^d^280 patients with reported data.

**Table 2:** Relationship between baseline patient characteristics and AB subgroups.

	AB = 0 (N = 176)	AB > 0 (N = 99)	*p* value^*∗*^
	n (%), Mean ± SD	n (%), Mean ± SD	
Patient characteristic			

Male gender	142 (81)	87 (88)	0.134

Age (years)	65.1 ± 10.8	67.5 ± 10.1	0.071

Body mass index (kg/m^2^)	27.5 ± 4.9	27.9 ± 4.9	0.455

NYHA functional class			0.003
NYHA ≤ II	95 (54)	37 (37)	
NYHA ≥ III	26 (15)	31 (31)	
No heart failure / not evaluated	55 (31)	31 (31)	

LVEF (%)	36.6 ± 13.6	33.1 ± 12.2	0.047

Secondary prevention indication	93 (53)	50 (51)	0.802

History of atrial fibrillation	23 (13)	40 (40)	< 0.001

Ischemic	115 (65)	66 (67)	0.791

Diabetes mellitus	47 (27)	23 (23)	0.665

Renal insufficiency	47 (27)	26 (26)	1.000

Sinus bradycardia	29 (17)	14 (14)	0.730

Cardiomympathy	99 (57)	57 (59)	0.798

Medication			

ACE inhibitor / angiotensin-antagonist	142 (81)	88 (89)	0.123

Amiodarone	40 (23)	24 (24)	0.882

Beta-blocker	152 (87)	87 (88)	0.853

Ca-antagonist	18 (10)	10 (10)	1.000

Spironolactone	57 (33)	36 (36)	0.596

Other diuretic	115 (66)	69 (70)	0.592

*Abbreviations.* NYHA: New York Heart Association; LVEF: left ventricular ejection fraction; ACE: angiotensin-converting enzyme; AB: atrial arrhythmia burden.

AB during follow-up was assessed as AB > 0 never occurring (AB = 0) vs. AB > 0 occurring at least once (AB > 0) during follow-up. Only subjects with at least 45 records during follow-up were included in this assessment. Therefore, AB subgroups do not sum up to the total of 283 subjects.

^*∗*^ P-value from t-test (numeric variables) or Fisher's exact test (categorical variables) for difference between AB subgroups.

**Table 3:** Allocation of patients with device-based recorded AF and history of AF.

	AB = 0	AB > 0	
History AF -	152	59	211
History AF +	23	40	63

Sum	175	99	274

*Abbreviations.* AB: atrial arrhythmia burden; AF: atrial fibrillation.

## Data Availability

The data used to support the findings of this study are not publicly available. They are restricted by patients privacy policy defined in the informed consent and the data protection law.
